# Barriers to the implementation of preconception care guidelines as perceived by general practitioners: a qualitative study

**DOI:** 10.1186/1472-6963-13-36

**Published:** 2013-01-31

**Authors:** Danielle Mazza, Anna Chapman, Susan Michie

**Affiliations:** 1 Department of General Practice, Monash University, Building 1, 270 Ferntree Gully Road, Notting Hill, VIC 3168, Australia; 2 Research Department of Clinical, Education and Health Psychology, University College London, London, UK

**Keywords:** Preconception care, Focus groups, Family practice, Practice guideline, Barrier analysis

## Abstract

**Background:**

Despite strong evidence of the benefits of preconception interventions for improving pregnancy outcomes, the delivery and uptake of preconception care and periconceptional folate supplementation remain low. General practitioners play a central role in the delivery of preconception care. Understanding general practitioners’ perceptions of the barriers and enablers to implementing preconception care allows for more appropriate targeting of quality improvement interventions. Consequently, the aim of this study was to examine the barriers and enablers to the delivery and uptake of preconception care guidelines from general practitioners’ perspective using theoretical domains related to behaviour change.

**Methods:**

We conducted a qualitative study using focus groups consisting of 22 general practitioners who were recruited from three regional general practice support organisations. Questions were based on the theoretical domain framework, which describes 12 domains related to behaviour change. General practitioners’ responses were classified into predefined themes using a deductive process of thematic analysis.

**Results:**

Beliefs about capabilities, motivations and goals, environmental context and resources, and memory, attention and decision making were the key domains identified in the barrier analysis. Some of the perceived barriers identified by general practitioners were time constraints, the lack of women presenting at the preconception stage, the numerous competing preventive priorities within the general practice setting, issues relating to the cost of and access to preconception care, and the lack of resources for assisting in the delivery of preconception care guidelines. Perceived enablers identified by general practitioners included the availability of preconception care checklists and patient brochures, handouts, and waiting room posters outlining the benefits and availability of preconception care consultations.

**Conclusions:**

Our study has identified some of the barriers and enablers to the delivery and uptake of preconception care guidelines, as perceived by general practitioners. Relating these barriers to a theoretical domain framework provides a clearer understanding of some of the psychological aspects that are involved in the behaviour of general practitioners towards the delivery and uptake of preconception care. Further research prioritising these barriers and the theoretical domains to which they relate to is necessary before a methodologically rigorous intervention can be designed, implemented, and evaluated.

## Background

Pregnancy is ‘a window of opportunity’ for promoting positive health behaviours because it is a time when women are more inclined to give up unhealthy habits [1]. While women realise the importance of optimising their health before pregnancy [2], many studies have shown that women of reproductive age demonstrate low levels of knowledge and behaviour related to preconception care (PCC) [2-6]. In one study, it was reported that less than 50% of women supplement their diet with folate during the periconception period [3] even though there is strong evidence that folate supplementation reduces the risk of neural tube defects (NTDs) [7].

National and international guidelines outlining evidence-based recommendations for the delivery and uptake of PCC have been published [8-11]. These recommendations focus on medical risk factors such as infection, immunisation status, previous adverse pregnancy outcomes, and patient lifestyle (eg, smoking, nutrition, alcohol, and physical activity). The delivery of PCC is generally achieved in a primary health care setting, but many women find that general practitioners (GPs) rarely discuss the availability of and need for PCC [12]. This assertion is further supported by a study which reported that only 53% of women who had heard of folate reported doing so from a GP or obstetrician [3]. Therefore, there is a need to understand why there is inadequate delivery of PCC guidelines by GPs.

Implementation researchers have emphasised that the identification of theoretical domains that are causally related to behaviour allows for the appropriate targeting of interventions for increasing guideline implementation [13-18]. A consensus of experts has identified a set of theoretical domains that may be considered when designing strategies to improve the implementation of evidence-based practice [19]. This set of domains has been used previously to understand implementation difficulties in different health care settings such as mental health care [20], primary health care [21], intensive care [22], and chiropractic care [23]. Theoretical domains have also been used to identify the barriers and enablers to careful hand hygiene as perceived by nurses and hospital administrators [24]. Therefore, theoretical domains can potentially be used to identify aspects of GPs’ behaviour which can be targeted for intervention to improve the delivery and uptake of PCC guidelines.

Our previous study examined women’s views on the barriers and enablers to the delivery and uptake of PCC [12], but there is little information on the views of GPs on the barriers and enablers to the delivery and uptake of PCC guidelines. Consequently, the aim of this study was to examine GPs’ views on the barriers and enablers to the delivery and uptake of PCC guidelines issued by the Royal Australian College of General Practitioners (RACGP) [25] using theoretical domains related to behaviour change.

## Methods

### Study participants

This study was approved by the Monash University Standing Committee on Ethics in Research Involving Humans and informed written consent was obtained from all participants. All participant details were de-identified to maintain confidentiality. To obtain a range of opinions that reflected diverse practice settings, purposive sampling using letters of invitation, advertisements in member newsletters, and faxes was used to recruit GPs through three regional general practice support organisations (two metropolitan and one rural). The Index of Relative Socio-Economic Disadvantage [26] was used to categorise practice postcodes from urban areas as either high or low socioeconomic status (SES). A total of 22 GPs (13 female and 9 male) participated in our study, with 10 GPs from low SES practices, 7 GPs from high SES practices, and 5 GPs from rural practices.

### Data collection

A total of three focus group interviews (one for each regional general practice support organisation) were conducted between October and November 2007. Focus group interviews were conducted because these were deemed to be most effective for engaging with busy GPs. To maintain consistency, all focus groups were conducted by the same facilitator (DM) and each session was 90 minutes in duration. The schedule of questions for the focus group (Table 1) was based on the theoretical domain framework developed by Michie et al. [19]. This framework outlines 12 key theoretical domains related to behaviour change (Table 2) and can be used to identify which domains are likely to be the best explanations of implementation problems. In this study, each domain was linked to a set of questions which were used to explore the delivery and uptake of PCC guidelines by GPs, as outlined in the RACGP Guidelines for Preventive Activities in General Practice [25]. The facilitator chose which set of questions to ask and the line of questioning was modified according to whether barriers and enablers were elicited for each domain.

**Table 1:** Schedule of questions for focus groups and the corresponding theoretical domains

**Domain**	**Interview questions**
Knowledge	· Do you know about the Preventive Activities Before Pregnancy (PABP) recommendations that are outlined in the “Guidelines for preventive activities in general practice” produced by RACGP?
	- If yes, what is your understanding of the recommendations?
	- If no, the recommendations are [description of PABP recommendations and a copy of the recommendations provided to participants]
Skills	· How do you usually deliver the PABP recommendations?
	· Can you give an example of how you have delivered the PABP recommendations?
	- What aspects of the PABP recommendations do you usually deliver?
Social/professional role and identity	· What are your views about the PABP recommendations in general?
	· Do you think it is an appropriate part of your job to be following these recommendations?
Beliefs about capabilities	· How difficult or easy is it for you to deliver the PABP recommendations?
	· What problems have you encountered?
	· What would help you to overcome these problems?
	· How confident are you that you can deliver the PABP recommendations despite the difficulties?
Beliefs about consequences	· What are the advantages of delivering the PABP recommendations to patients?
	· What are the disadvantages?
	· Would you say that the benefits outweigh the costs?
Motivation and goals	· How much do you want to deliver the PABP recommendations?
	· How much do you feel you should deliver the PABP recommendations?
	· Are there other things you want to do or achieve that interfere with the delivery of the PABP recommendations?
Memory, attention and decision processes	· Do you think to deliver the PABP recommendations?
	· How much attention do you have to pay to deliver the PABP recommendations?
	· What are some reasons for deciding not to deliver PABP recommendations?
Environmental context and resources	· To what extent do physical or resource factors facilitate or hinder you in delivering the PABP recommendations?
	· Are there competing tasks and time constraints that impact the delivery of PABP recommendations?
	· Do you have the necessary resources available to you to deliver the PABP recommendations?
Social influences	· To what extent do social influences of peers, practice staff etc.… facilitate or hinder you in delivering the PABP recommendations?
	· Do you observe other peers and role models delivering PABP recommendations?
Emotion	· Do you think that any emotional factors influence whether PABP recommendations are delivered?
Behavioural regulation	· Are there procedures or ways of working that encourage you to deliver the PABP recommendations?
Nature of behaviour	· What might need to be done differently in order to increase delivery of PABP recommendations?
	· What would you do differently?

**Table 2:** Theoretical domains and their component constructs

**Domains**	**Constructs**
1. Knowledge	Knowledge
	Knowledge about condition/scientific rationale
	Schemas, mindsets and illness representations
	Procedural knowledge
2. Skills	Skills
	Competence/ability/skill assessment
	Interpersonal skills
	Coping strategies
3. Social/professional role and identity	Identity
	Professional identity/boundaries/role
	Group/social identity
	Social/group norms
	Alienation/organisational commitment
4. Beliefs about capabilities	Self-efficacy
	Control – of behaviour and material and social environment
	Perceived competence
	Self-confidence/professional confidence
	Empowerment
	Self-esteem
	Perceived behavioural control
	Optimism/pessimism
5. Beliefs about consequences	Outcome expectancies
	Anticipated regret
	Appraisal/evaluation/review
	Consequences
	Attitudes
	Contingencies
	Reinforcement/punishment/consequences
	Incentives/rewards
	Beliefs
	Unrealistic optimism
	Salient events/sensitisation/critical incidents
	Characteristics of outcome expectancies – physical, social, emotional; sanctions/rewards, proximal/distal, valued/not valued, probable/improbable, salient/not salient, perceived risk/threat
6. Motivation and goals	Intention; stability of intention/certainty of intention
	Goals (autonomous, controlled)
	Goal/target setting
	Goal priority
	Intrinsic motivation
	Commitment
	Distal and proximal goals
	Transtheoretical model and stages of change
7. Memory, attention and decision processes	Memory
	Attention
	Attention control
	Decision making
8. Environmental context and resources	Resources/material resources (availability and management)
	Environmental stressors
	Person x environment interaction
	Knowledge of task environment
9. Social influences	Social support
	Social/group norms
	Organisational development
	Leadership
	Team working
	Group conformity
	Organisational climate/culture
	Social pressure
	Power/hierarchy
	Professional boundaries/roles
	Management commitment
	Supervision
	Inter-group conflict
	Champions
	Social comparisons
	Identity; group/social identity
	Organisational commitment/alienation
	Feedback
	Conflict – competing demands, conflicting roles
	Change management
	Crew resource management
	Negotiation
	Social support: personal/professional/organisational, intra/interpersonal, society/community
	Social/group norms: subjective, descriptive, injunctive norms
	Learning and modelling
10. Emotion	Affect
	Stress
	Anticipated regret
	Fear
	Burn-out
	Cognitive overload/tiredness
	Threat
	Positive/negative affect
	Anxiety/depression
11. Behavioural regulation	Goal/target setting
	Implementation intention
	Action planning
	Action planning
	Self-monitoring
	Goal priority
	Generating alternatives
	Feedback
	Moderators of intention-behaviour gap
	Project management
	Barriers and facilitators
12. Nature of behaviours	Routine/automatic/habit
	Breaking habit
	Direct experience/past behaviour
	Representation of tasks
	Stages of change model

### Data analysis

Data from focus groups were audio-taped and transcribed verbatim. To familiarise themselves with the data, two of the authors (DM and AC) read all of the transcripts twice. To increase rigour, both authors independently coded each transcript, line by line. The data was then organised and analysed using NVivo 7 software [27]. A deductive process of thematic analysis [28,29] was used to classify responses within themes, and the theoretical domains previously described (Table 2) were used as the coding framework. Our results revealed good inter-rater reliability (76%). For instances where coding differed, an agreement of interpretation was reached through meetings with the project advisory group, which included content and methodological experts.

## Results

We found that the barriers and enablers to the delivery and uptake of PCC guidelines, as perceived by GPs, were primarily related to four theoretical domains: (1) beliefs about capabilities; (2) motivations and goals; (3) environmental context and resources; and (4) memory, attention, and decision making. Statements which accurately depicted the theme for each theoretical domain were chosen accordingly. Theoretical domains that were found to not be relevant included emotion, social/professional role and identity, and skills.

### Beliefs about capabilities

GPs felt that they did not have the opportunity to deliver the PCC guidelines as women often did not present at the preconception stage. GPs also stated that they were often unaware of a woman’s intention to conceive, or that women only presented when they were already pregnant.

“You’ve got to motivate them to actually come in; my main barrier is that they don’t come.” (Female GP, High SES)

“It’s very rare for people to come in and say that they’re planning to have a baby. Most of them come when they are already pregnant.” (Male GP, High SES)

GPs also stated that increased awareness in women of both the availability and the importance of PCC would facilitate the delivery of PCC guidelines.

### Motivation and goals

A number of GPs stated that spina bifida and NTDs were too rare to warrant specific action. This view was reflected by the fact that few of the GPs had ever seen a case of NTD. They were also of the opinion that folate isn’t 100% effective in preventing NTDs, and that even if they did discuss the use folic acid during early pregnancy, their efforts may not be worthwhile because NTDs were still a possibility.

“… even if everyone did take folate, there will still be neural tube defects, it will never be eradicated.” (Female GP, Low SES)

GPs also frequently stated that they did not raise PCC with women of reproductive age because of other competing preventive care priorities. Examples of other preventive care priorities included chlamydia screening, pap smears, and cervical cancer vaccination. Some GPs preferred to spend more time addressing issues such as alcohol and smoking as they could see more potential benefits.

“It’s a big consultation and there is a lot of competing preventive health at the moment.” (Female GP, Low SES)

### Environmental context and resources

Overall, the biggest barrier to the delivery of PCC as perceived by GPs was the time limits on consultations. GPs pointed out that the delivery of PCC guidelines would take longer than a standard consultation, and that trying to cover all of the issues related to PCC was a real challenge. They found it particularly problematic when PCC issues were raised at the end of a consultation for another issue.

“There is a problem with adding in more preventive type consultations in practices that are stretched to the limit. I don’t know if we can physically do it….” (Male GP, Rural)

“The main commodity is time, and all of us are stretched at the moment so by taking on this additional work, we don’t know where to fit it in.” (Male GP, High SES)

More importantly, both rural and low SES groups of GPs felt that patients were not willing to spend more time and money on attending multiple consultations dedicated to PCC.

“There is also the cost as well, because they won’t want to pay for that.” (Female GP, Rural)

Another major barrier identified by GPs was the lack of both GP and patient resources (eg, patient information sheets, evidence-based websites) for PCC. The majority of resources utilised by GPs were patient information sheets produced by pharmaceutical companies. Consequently, GPs expressed a need for resources to be produced by more credible, unbiased organisations.

Another barrier that was mentioned by GPs was the limited access to individual GPs, particularly for rural patients. There was some concern that not all GPs were willing to deliver PCC, and, consequently, there could be considerable delay to see those that do.

“A problem is that in our practice, there is a lot of doctors who don’t do prenatal care and, if they want to see me, it’s a long wait.” (Female GP, Rural)

“Another problem is that I’m not always there, which makes it more difficult if they want to see me.” (Female GP, Low SES)

One final barrier that was identified was the potential increase in burden on clinics if the number of PCC consultations was increased. GPs commonly believed that this may result in sick patients getting limited access to clinics. GPs stated that they often pushed themselves to make additional time for patients who were unwell, but they were not necessarily willing to keep adding more time to consultations for opportunistic or preventive issues.

*“If I book for half hour to talk about this, I’ve got other patients who are actually sick and are coming with hepatitis, influenzas* etc.*… and I have to make time for them, so I’m not going to allow more time for something that is preventive or an opportunistic thing.” (Male GP, Low SES)*

### Memory, attention and decision making

A perceived enabler identified by GPs was the availability of PCC checklists to ensure that the entire PCC guideline is discussed. Patient brochures, handouts, and waiting room posters outlining the benefits and availability of PCC consultations were also viewed as beneficial.

“I like the idea of having a checklist of things so when people come in you can go over everything.” (Male GP, Low SES)

## Discussion

Using the theoretical domain framework, our study has identified some of the barriers and enablers to the delivery and uptake of PCC guidelines, as perceived by GPs. The biggest barrier we identified was the time constraints faced by GPs – they simply felt that there was not enough time to deliver PCC in a standard consultation. Other barriers to the delivery of PCC guidelines were the lack of women presenting at the preconception stage, other competing preventive care issues, the availability of and access to GPs who deliver PCC, the cost associated with extending consultations to include PCC, and the lack of resources for assisting in the delivery of preconception care guidelines. Perceived enablers to the delivery and uptake of PCC guidelines included the availability of PCC checklists as well as patient brochures, handouts, and waiting room posters outlining the benefits and availability of PCC consultations.

The results of our study highlight the need for developing interventions that respond to concerns from GPs about their capability to deliver PCC when faced with time constraints. The availability of a checklist may prove useful for GPs as it will ensure that all aspects of the PCC guidelines are discussed with patients, even when time is limited. There is also the potential for PCC to be delivered by a practice nurse or for a risk screen to be undertaken online by patients prior to a consultation. Both options could potentially remove some of the burden on GPs without compromising the delivery of PCC to patients.

The lack of women presenting at the preconception stage also contributes to the difficulty in delivering PCC guidelines. Interventions should focus on increasing patient awareness of the need for and availability of PCC. The lack of women presenting specifically for PCC results in GPs being deprived of the opportunity to devote a consultation to this purpose. As a consequence, PCC issues are often raised at the end of the consultation where they may compete with other preventive care issues. Dealing with PCC in an “opportunistic” way is problematic because non-attendees and those who are most in need may inadvertently be denied access to PCC. A shift in emphasis is required that extends women’s awareness to attend for antenatal care at the preconception stage. Some have even suggested extending PCC to the family planning setting whilst acknowledging the difficulty of talking to a predominantly young clientele about preparing for pregnancy at a time when they are presenting for contraceptive services [30].

GPs also face difficulties with prioritising PCC together with other preventive care issues. GPs are unsure about which of the many preventive care issues are most important to address. This issue has potential implications for those attempting to implement other preventive care guidelines related to cancer screening, chronic disease prevention, and screening for sexually transmitted diseases such as chlamydia. The problem may lie with the opportunistic way in which preventive care is delivered by GPs. A potential solution to this problem may be to schedule all consultations related to preventive care so that the burden of deciding which preventive care issue is most important is removed from the GPs. Alternatively, GPs could systematically identify and recall patients who are in need of preventive care.

Both the cost of and access to preventive services in low SES and rural areas were viewed by GPs in our study as problematic to the delivery of PCC guidelines. These issues are often discussed in literature from the US, where insurance coverage for women of reproductive age living in low SES and rural areas is a problem [31]. Inadequate access to PCC may be overcome by work-role substitution, where practice nurses undertake the delivery of PCC. Also, the availability of a financial incentive specifically for PCC would overcome the cost of accessing PCC, which is especially relevant for women living in low SES areas.

In our study, GPs also mentioned the need for GP and patient resources to facilitate the delivery of PCC guidelines. Specifically, GPs thought that the availability of checklists would ensure that the entire guideline is discussed during a consultation. Guidelines often have a multitude of recommendations and, therefore, GPs need assistance with resources and time management strategies to enable them to deliver the recommendations in a more holistic way. Our findings are consistent with a previous study that examined the barriers to counselling women of childbearing age on the potential risks of birth defects when using certain medications during pregnancy. In this study, primary care providers expressed a desire for resources such as patient information materials, electronic decision support tools, and clinical care systems that routinely assess patients' pregnancy risk, which they believed would help counsel patients about the teratogenic risks associated with certain medications [32]. Another study also highlighted the need for patient information leaflets to support GPs in the delivery of PCC to women with diabetes, especially when there are time constraints [33]. Consequently, potential interventions for improving the delivery of PCC guidelines should also focus on providing tools and resources to assist GPs in delivering the content and evidence base of the guidelines.

Our study is limited by the relatively small number of GPs involved and the fact that they were recruited from only one state in Australia. Despite this, the barriers and enablers (and, therefore, the corresponding theoretical domains) identified by GPs were consistent across the three regional general practice support organisations. Although GPs in low SES and rural groups already consult ethnically diverse populations, our study could have been further strengthened by recruiting GPs who work in locations with high indigenous populations. Indigenous populations have proportionately higher rates of perinatal morbidity and mortality relative to the rest of the Australian population. Therefore, the views of those GPs on the barriers and enablers to the delivery and uptake of PCC would have provided an interesting comparison with our data.

The results of our study are also limited by the qualitative design. While focus groups are suitable for exploring common experiences, they may increase the conformity of responses. Because the GPs in our study volunteered to participate, they may also represent a subgroup of GPs who have a higher degree of interest in PCC compared with other GPs, thus limiting the generalisability of our findings. The barriers reported by GPs may also be different to those observed in real practice. For example, a lack of time to deliver PCC may, in reality, reflect a lack of motivation. Conducting a similar study on a larger scale or incorporating quantitative methodologies may provide a greater understanding of the issues raised in our study.

## Conclusion

Our study has identified some of the barriers and enablers to the delivery and uptake of PCC, as perceived by GPs. Further research is necessary to determine which of these should be targeted or prioritised for intervention. Consideration must also be given to the views of women on the barriers and enablers to the delivery and uptake of PCC [12]. Understanding the views of both women and GPs as well as the theoretical basis for changing their behaviour will be essential when designing effective implementation strategies for improving the delivery and uptake of PCC. These strategies may also need to consider the role practice nurses and other health professionals may have in facilitating better uptake of PCC, especially among high-risk patients who should be actively targeted. Promotional materials and letters of invitations from GPs advising patients of the availability of and the need for preconception care could also be used to increase the uptake of PCC. Given the potential for evidence-based PCC to reduce maternal and neonatal morbidity and mortality, it is essential that effective strategies are put in place to deliver evidence-based PCC guidelines. Therefore, the information gathered by our study provides a foundational step for designing a methodologically rigorous intervention [34] for improving the implementation of PCC guidelines.

## Abbreviations

GP: General practitioner; NTD: Neural tube defect; PCC: Preconception care; RACGP: Royal Australian college of general practitioners; SES: Socioeconomic status.

## Competing interests

The authors declare that they have no financial or non-financial competing interest.

## Authors’ contributions

DM conceived the study, DM and AC drafted the paper, and SM contributed to the writing and review of the manuscript. All authors read and approved the final manuscript.

## Pre-publication history

The pre-publication history for this paper can be accessed here:

http://www.biomedcentral.com/1472-6963/13/36/prepub
